# Comparison of Parameter Threshold Combinations for Diffusion Tensor Tractography in Chronic Stroke Patients and Healthy Subjects

**DOI:** 10.1371/journal.pone.0098211

**Published:** 2014-05-22

**Authors:** Martin Domin, Sönke Langner, Norbert Hosten, Martin Lotze

**Affiliations:** 1 Functional Imaging Unit, Center for Diagnostic Radiology and Neuroradiology, University Medicine, Greifswald, M/V, Germany; 2 Center for Diagnostic Radiology and Neuroradiology, University Medicine, Greifswald, M/V, Germany; INSERM U894, Centre de Psychiatrie et Neurosciences, Hopital Sainte-Anne and Université Paris 5, France

## Abstract

**Background:**

Although quantitative evaluation of diffusion tensor imaging (DTI) data seemed to be extremely important for clinical research its application is under debate. Besides fractional anisotropy (FA) the quantitative comparison between hemispheres of the number of fibers reconstructed by means of diffusion tensor tractography (DTT) is commonly used. However, the tractography-related parameters FA, minimum tract length (LENGTH) and the angle between two contiguous tracking steps (ANGLE) are inconsistently applied. Using 18 combinations we tested for the influence of parameter thresholds on the amount of reconstructed fibers for the posterior pyramidal tract in both hemispheres in order to obtain meaningful thresholds for DTT.

**Results:**

In 14 chronic stroke patients with unilateral lesions of the pyramidal tract around the height of the internal capsule and considerable motor deficits a 3-way repeated-measures ANOVA showed a significant interaction between the effects of FA and ANGLE level on reconstructed fiber lateralization, *F* (2.9, 37.67) = 3.01, *p* = 0.044, and a significant main effect FA, *F* (1.4, 18.1) = 11.58, *p* = 0.001. Post-hoc pairwise comparisons showed that this interaction was completely driven by FA. In 22 right-handed healthy subjects no significant interactions or main effects could be found.

**Conclusion:**

The parameter threshold combinations with highest FA showed highest effect. ANGLE and LENGTH insofar influenced the lateralization effect when selected as liberal as possible, short LENGTH and large ANGLE thresholds. The DTT approach should be used with great care since results are highly dependent on the thresholds applied.

## Background

There is a high clinical demand for quantitative evaluation of diffusion tensor imaging (DTI). Especially in neurology a quantitative comparison of white matter tracts between the affected and the unaffected side after stroke or a comparison of a patient group with a group of healthy controls is frequently asked for. A quantitative comparison might be perfect to identify and describe individual or group damage of different white matter tracts in several groups of patients as for instance those with amyotrophic lateral sclerosis, multiple sclerosis or traumatic brain injury. However, the application of quantitative methods is a subject of debate.

Quantitative assessment of cerebral white matter tracts is based on the diffusion of water in neural fibers. This anisotropic diffusion is restricted to the dense packing of axons and axonal membranes [1]. The ratio of unrestricted and restricted water diffusion in human white matter can be expressed by fractional anisotropy (FA), calculated by means of the diffusion tensor [2] as a scalar value in the range of zero (total isotropic) to one (total anisotropic). FA of the pyramidal tract after subcortical stroke shows high associations with clinical data such as motor scores [3]–[5] or demyelating disease [6]. Even more, subtle differences between white matter tracts of the dominant and non-dominant hemisphere in healthy subjects might be quantitatively characterized with FA (for the anterior limb of the internal capsule: [7]). However, this finding is controversially discussed [8].

In contrast, diffusion tensor tractography (DTT) reconstructs an approximate representation of white matter structure. The same diffusion tensor, which is calculated from diffusion-weighted MRI data, not only contains the magnitude of water diffusion but also the direction. By means of this quantifiable directionality the reconstruction of large white matter fiber tracts is possible. DTT is well established e.g. in neurosurgery planning and intraoperative updates [9]–[12], in the study of anatomical connectivity [13]–[15] and in the parcellation of cortical as well as subcortical structures [16]–[18]. Studies examining the validity of Diffusion Tensor Tractography found good visual and volumetric similarities of DTT-reconstructed fiber tracts with histology and classical dissections of human and animal brains [19]–[21]. Although these studies show that DTT can be used to evaluate structural differences in white matter of the human brain, great care has to be exercised when selecting the diffusion tensor model as the basis for tractography. Among several models interpreting the diffusion profile as a surrogate for local white matter structure the diffusion tensor model is the least powerful one. The drawbacks of this model such as the inability to resolve crossing or kissing fibers are described elsewhere [22], [23]. Despite of the partially severe weaknesses DTT is frequently used due to its low complexity compared to other models and its high availability in most MRI scanners and in a considerable amount of scientific diffusion-MRI-related software packages.

We decided to perform a systematical investigation of the influence of the main tracking parameters on the DTT results. For parameter thresholds we investigated the fractional anisotropy (FA), the angle between two contiguous tracking steps (ANGLE) and the required minimum reconstructed fiber length (LENGTH). We intended to find a parameter combination that produces the largest effect for possible inter-hemispheric differences in the posterior limb of the internal capsule. In addition, inter-hemispheric differences were calculated for the FA values. We used two different subject groups for these comparisons. A strong inter-hemispheric effect was expected for a group of 14 patients who experienced subcortical stroke with relevant unilateral hand motor impairment on average 82 weeks before diffusion-weighted imaging.

A moderate to undetectable effect was expected for a group of 22 right-handed healthy participants. For those we expected higher FA and DTT values for the dominant hemisphere in comparison to the non-dominant hemisphere.

## Results

### FA values

The evaluation of inter-hemispheric differences for fractional anisotropy revealed a higher mean FA value (*t* (13) = −2.56; *p* = 0.024) for the unaffected hemisphere in the patient group and a higher mean FA value for the dominant hemisphere for the group of healthy controls (*t* (21) = 2.27; *p* = 0.034).

### DTT parameter threshold combinations

A three-factor repeated-measures Analysis of Variance (ANOVA) was conducted to evaluate the effects of the DTT parameter thresholds on the reconstructed fibers lateralization index. The three independent variables in this study were FA, ANGLE and LENGTH. The reconstructed fibers lateralization index was used as the dependent variable.

#### Patients

**Figure 1:**
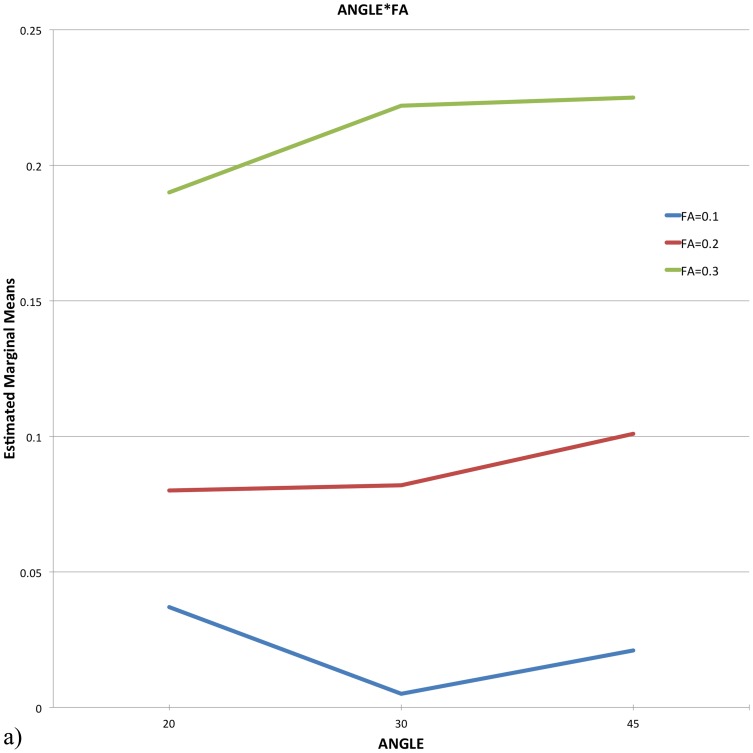
Repeated-measures ANOVA: Interaction graphs for ANGLE*FA. The graph illustrates that the FA threshold is essential for modulating the lateralization effect.

**Figure 2:**
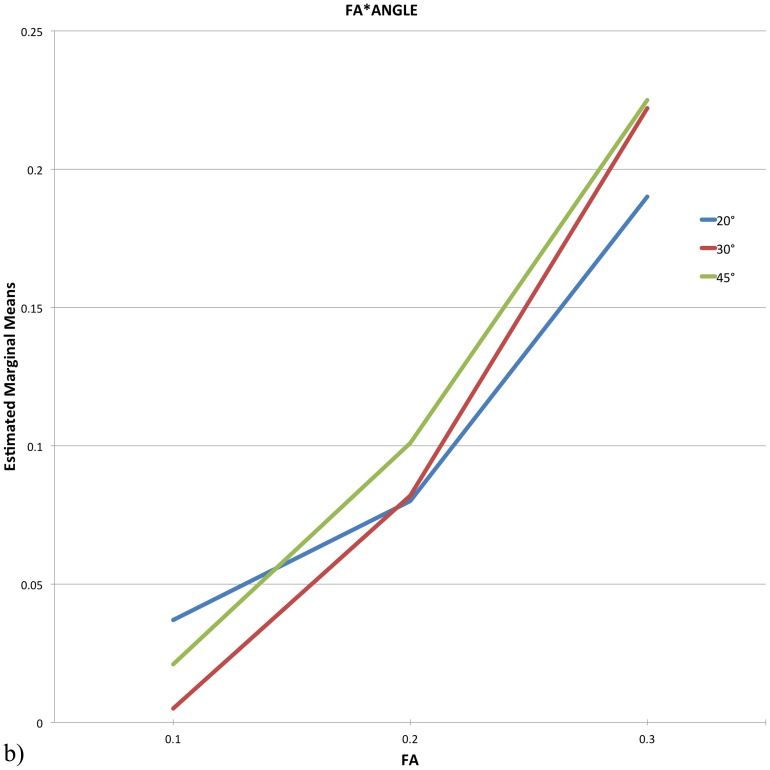
Repeated-measures ANOVA: Interaction graphs for FA*ANGLE. The interaction graph, similar to Figure 1, but in the opposite way, again illustrates the crucial influence of the FA threshold.

The results of the three-way repeated measures ANOVA indicated a significant main effect for FA (*F* (1.4, 18.1) = 11.581, *p* = 0.001) and a significant interaction of FA and ANGLE on the reconstructed fibers lateralization index, *F* (2.9, 37.67) = 3.01, *p* = 0.044 (see Figure 1 and Figure 2 for interaction).

The post-hoc pairwise comparisons for the main effect FA showed, that the reconstructed fibers lateralization index for FA = 0.3 was significantly larger than for FA  = 0.2 (*p*<0.008) or for FA  = 0.1 (*p*<0.009).

Additional post-hoc pairwise comparisons were conducted to examine the influence of ANGLE and FA on the interaction. For ANGLE  = 30° they revealed a significantly larger reconstructed fibers lateralization index for FA  = 0.3 than for FA  = 0.2 (*p*<0.009) as well as for FA  = 0.1 (*p*<0.007). For ANGLE  = 45° the follow-up comparisons showed a similar result, the reconstructed fibers lateralization index for FA  = 0.3 again was significantly larger than for FA  = 0.2 (*p*<0.009) or for FA  = 0.1 (*p*<0.012).

#### Healthy subjects

The repeated-measures ANOVA showed no significant interactions or main effects.

### Effect sizes

**Figure 3:**
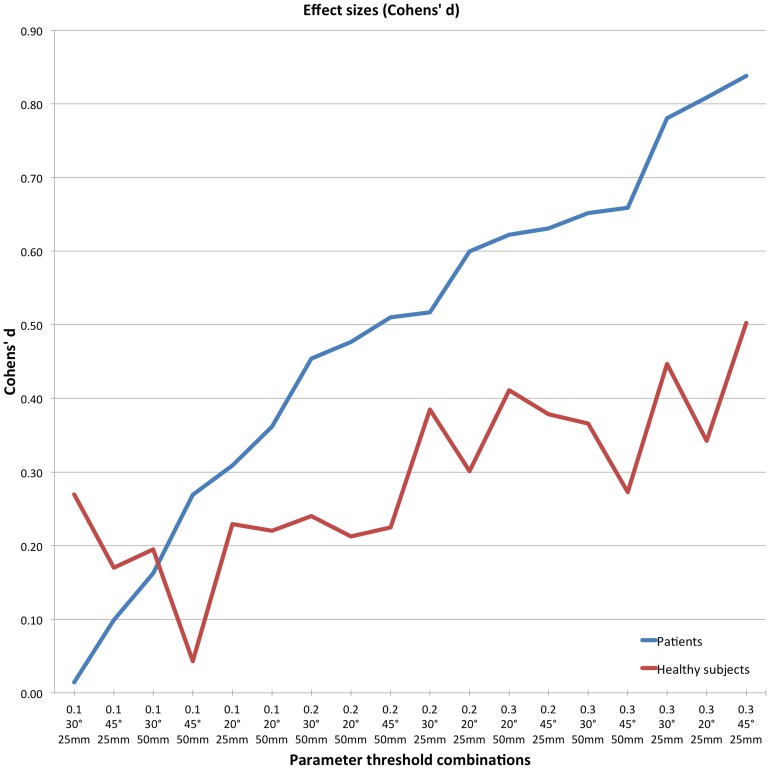
Effect sizes of parameter threshold combinations. The plot shows the effect size of each tested parameter threshold combination utilizing Cohens' d. The data were sorted by the effect sizes of the patient group.

Paired t-tests and the following calculation of the effect size measure Cohens' d were conducted for patients and healthy subjects to identify the threshold parameter combination with the highest effect in regards to the difference between hemispheres. Although the group of healthy subjects did not show significant main effects or interactions in the repeated-measures ANOVA, the t-statistics for this group were used for display reasons. The results of the t-statistics are shown in Table 1. Cohens' d was used to calculate effect sizes (plotted in Figure 3), depicting a small statistical effect with about d = 0.2, a medium with d = 0.5, and a large effect with d = 0.8. For both the patients (d = 0.84) and the healthy subjects group (d = 0.5) the same combination (FA  = 0.3, ANGLE  = 45°, LENGTH  = 25 mm) showed the largest effect.

**Table 1:** t-values and Cohens'd of parameter threshold combinations for patients and healthy subjects.

Length	Angle	FA	Patient group		Healthy subjects group	
			t(13)	Cohens' d	t(21)	Cohens' d
25 mm	20°	0.1	−1.16	0.31	−1.08	0.23
25 mm	20°	0.2	−2.24	0.60	−1.41	0.30
25 mm	20°	0.3	−3.03	0.81	−1.61	0.34
25 mm	30°	0.1	0.05	0.01	−1.26	0.27
25 mm	30°	0.2	−1.93	0.52	−1.81	0.39
25 mm	30°	0.3	−2.92	0.78	−2.10	0.45
25 mm	45°	0.1	−0.37	0.10	−0.80	0.17
25 mm	45°	0.2	−2.36	0.63	−1.78	0.38
25 mm	45°	0.3	−3.14	0.84	−2.36	0.50
50 mm	20°	0.1	−1.35	0.36	−1.03	0.22
50 mm	20°	0.2	−1.78	0.48	−1.00	0.21
50 mm	20°	0.3	−2.33	0.62	−1.93	0.41
50 mm	30°	0.1	−0.61	0.16	−0.91	0.19
50 mm	30°	0.2	−1.70	0.45	−1.13	0.24
50 mm	30°	0.3	−2.44	0.65	−1.72	0.37
50 mm	45°	0.1	−1.01	0.27	−0.20	0.04
50 mm	45°	0.2	−1.91	0.51	−1.05	0.22
50 mm	45°	0.3	−2.47	0.66	−1.28	0.27

## Discussion

The present diffusion tensor imaging study aimed to investigate the influence of parameter thresholds on diffusion tensor tractography and to compare the combinations of these parameters with regards to the most significant and most effective difference between hemispheres in the human brain. When comparing different parameter thresholds for DTT we found highly different results depending on the thresholds used. Generally, FA seemed to be the most relevant parameter. Using a high FA threshold (in our study 0.3) resulted in significant inter-hemispheric differences between the DTT-values of the pyramidal tract of the affected and non-affected hemispheres in chronic stroke patients being left with a relevant paresis of their hand after subcortical damage. This result was confirmed and quantified by calculating the fractional anisotropy between both posterior limbs of the internal capsule.

We also tested for possible quantitative effects between the dominant and non-dominant hemisphere in healthy subjects. Again, the FA showed a significant inter-hemispheric difference with increased anisotropic diffusion for the dominant hemisphere. However, although the FA-values between the dominant and non-dominant hemisphere differed, DTT showed to be not sensitive enough to confirm these results. This finding is consistent with controversial reports about differences in pyramidal tract quantitative measures in healthy subjects such as the number of reconstructed fibers, tract volume and FA [7], [8].

Overall, these data support the opinion that FA is a more sensitive parameter for differentiating inter-hemispheric asymmetries than tractography based on the diffusion tensor approach. This might well be caused by essential differences of both parameters: whereas tractography is based on the tracing of the first principal eigenvector (PEV) of the diffusion tensor, FA depicts an indirect scalar directionality measure and is calculated by means of the eigenvalues of that same tensor. In linear algebra, the method of diagonalization is used to decompose a tensor into its eigenvalues and eigenvectors. In diffusion tensor imaging this method induces two independent alternatives of describing structural integrity in the human white matter. Fractional anisotropy describes the local, rotationally invariant directionality in a voxel of the diffusion tensor data set, independent from neighboring voxels. The tractography, on the opposite, calculates a path in a vector field. The signal-to-noise-ratio (SNR) or, more specifically, imaging noise considerably influences the calculation of the diffusion tensor and therefore the results of tensor diagonalization [24]. The influence on both eigenvalues and eigenvectors differs in a way that the eigenvalues could be over- or underestimated, whereas eigenvector noise leads to a random walk of calculated trajectories in simple fiber tracking schemes such as FACT [25], resulting in fast accumulation of uncertainties and deviations along the traced path and maybe leading to distorted fiber tract reconstructions. The overall effect that increased FA thresholds increase the effect size might be explained with a filtering process in a way that isotropic voxels are excluded from the tractography, leading to a removal of low anisotropy reconstructed fiber tracts, resulting in pronounced inter-hemispheric differences for the patients group. In our study a FA value of 0.3 showed the most significant results, so this could be seen as a recommended threshold for other researchers performing similar investigations of the internal capsule. However, it has to be kept in mind, that our method and the FA-thresholds with largest effect sizes might be valid only for the posterior limb of the internal capsule. In addition, the fractional anisotropy is dependent on the field strength of the MRI scanner [26], thus a recommendation has to be given with due care.

Even though the FA values between the dominant and non-dominant hemisphere in healthy subjects differed, low to medium effect sizes for the threshold combinations might have resulted in no relevant differences for the tensor tractography.

Since the patient and control group differed in gender this might have influenced our result. It had been reported before that women show differences in callosal white matter parameters in comparison to men [27]. We therefore avoided direct comparison between subject groups.

To improve data quality and signal-to-noise ratio the application of noise filters as proposed by [28]–[30] could be worth investigating. A systematic differentiation of the variation of noise reduction of DTI data is recommended for systematic comparisons for future work. Likewise, more sophisticated models like DSI (Diffusion spectrum imaging) [31], Q-Ball [32] or probabilistic approaches [33] for improved calculation of diffusion profiles and tractograms should be taken into consideration, which could reduce tensor model related issues like the missing abilities to reliably detect fiber crossing or kissing. At last, more advanced statistical approaches extending beyond simple t-statistics could improve results [34], [35].

Nevertheless, our data support the opinion that DTT can be used to evaluate structural differences in white matter of the human brain when applied with caution [22], [23]. In addition we systematically investigated the influence of the various thresholds reported in literature to be applied on DTT. Researchers should be aware that the results of tractography based on the diffusion tensor approach are highly dependent on the thresholds applied and FA values are first choice for quantifying diffusion of white matter tracts.

## Conclusions

We examined the dependence of DTT lateralization effects on tractography-related parameter thresholds. We found that the parameter threshold combinations with highest FA showed highest significance and effect size. Only extremely short LENGTH and large ANGLE thresholds were capable to influence the lateralization effect. Since the results of the diffusion tensor tractography are highly dependent on the thresholds applied, researchers planning to examine fiber tract asymmetries should exercise great care. Further studies of the influence of parameter thresholds on other white matter structures and artifact reduction such as more advanced diffusion models and noise-removal methods might be the next step for investigations.

## Methods

### Subjects

14 patients (mean age: 62 years; SD: 12.3 years; age range: 45–81years; 3 females, 11 males) with unilateral lesions in the internal capsule (mean lesion age: 82.1 weeks; SD: 28.3 weeks; lesion age range: 42.5–134.1 weeks), as verified in the T1-weighted high-resolution dataset were included into this comparison. Overall, they showed only a moderate impairment of paretic hand grip strength (affected hand: 31.7±8.5 kg; unaffected hand: 34.7±9.4 kg; t_13_ = 2.13, p = 0.028; one tailed). In our patients

Additionally, 22 healthy right-handed volunteers were examined (mean age 48.3 years; SD: 21.6 years; age range of 25–78 years; 17 female, 5 male) in order to evaluate possible hemispheric differences in the quantitative assessment of the pyramidal tract between hemispheres.

Participants in both groups provided written informed consent, which was approved by the Ethics Committee of the Medical Faculty of the University of Greifswald (Reg. Nr.: BB 51/07a).

### DTI measurements

DTI was conducted by using a 3 Tesla MRI-Scanner (Verio, Siemens, Erlangen, Germany) with a 32-channel head coil. The DTI-sequence used was a Siemens MDDW (Multi Directional Diffusion Weighting) sequence with the following parameter setup: matrix size: 128×128 native, FOV: 230 mm, voxel size: 1.8 mm isotropic, no slice gap, number of slices: 80, number of acquisitions (averages): 1, number of directions: 64, TR: 15300 ms, TE: 107 ms, total scan time: 16 min. Additionally, an anatomical 3D T1-weighted MPRage dataset was acquired. Matrix size: 256×256, voxel size: 1 mm isotropic, 176 slices, TE: 2.52 ms, TR: 2900 ms.

### Data processing

After file format conversion from native DICOM to NIFTI by dcm2nii (MRIcron), datasets underwent a standard preprocessing procedure as proposed by the FSL Analysis Group (Analysis Group, FMRIB, Oxford, UK) [36]. This included *eddy_correct* for eddy current correction and correction for subject motion using affine registrations to a reference volume, *bet* [37] as the tool of choice for skull stripping for improved co-registration and the tool *flirt* for linear (affine) co-registration [38]. At first, a linear co-registration was performed between the first volume of the diffusion data and the high resolution 3D-T1 volume. Another linear registration step was then performed between the 3D-T1 data set and the MNI template (MNI152 nonlinear 6^th^ generation). The tool *fnirt* was used to calculate a non-linear transformation into the MNI space in order to receive information for an inverse normalization for transposing masks. The non-linear step was calculated between the 3D-T1 and the MNI template to take advantage of the high resolution and tissue contrast in comparison to the lower resolution diffusion-weighted data set. The found registration transformations were combined using the tool *convertwarp*. For all following data evaluations we used the diffusion data within the individual space to avoid data modification by a normalization process. An inverse warp of the previously combined (non-)linear transformation into MNI space was calculated using the FSL tool *invwarp*. This inverse transformation was used to de-normalize the JHU-Whitematter-Labels, which contain all major white matter structures, particularly the posterior limb of the internal capsule in both hemispheres. This white matter label set was created in MNI space and is part of the FSL package. At last the gradient vectors used during the MRI measurement for the diffusion-weighting gradients were corrected in regards to the rotations being calculated during motion correction and linear co-registration.

### Tensor calculation, ROI placing and fiber tracking

JavaDTI, an in-house application, was used for data evaluation. The DTI datasets were loaded into the program and tensor calculation took place. As described in [39] the six components of the symmetric tensor matrix were calculated. To ensure positive semi-definite tensors, negative eigenvalues of the tensor matrix were reset to zero. The ROIs of the posterior limb of the internal capsule corresponding to the current data set were loaded into the program. The de-normalization of the white matter masks results in an “automatic” ROI placement, which improves comparability of results and in contrast to manually created ROIs drawn by a user no inter-rater bias has to be taken into account.

A review of a selection of publications using DTT showed an extensive range of chosen fiber tracking parameter thresholds [3], [8], [40]–[44]. For FA a range between 0.13 and 0.2 could be found, for the angle between two contiguous tracking steps a range from 27° to 41° and a minimum reconstructed fiber length threshold of 37.5 mm could be found [43]. Due to these findings we decided on the following parameter threshold values:

FA: 0.1, 0.2, 0.3
Angle between two contiguous tracking steps: 20°, 30°, 45°
Minimum length: 25 mm, 50 mm

**Figure 4:**
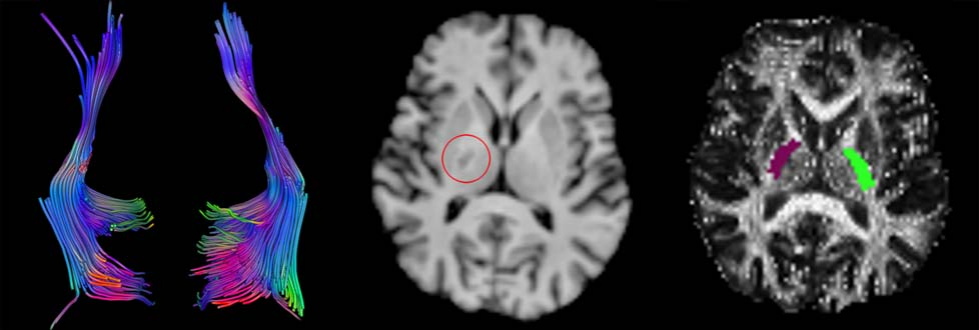
Diffusion tensor tractography in a stroke patient. The results of diffusion tensor tractography (left) in a patient who experienced subcortical stroke, causing relevant unilateral hand motor impairment. The lesion in the left posterior limb of the internal capsule is marked by a red circle on a T1-weighted anatomical slice image (middle), the de-normalized ROIs are shown as colored overlays on an FA slice image (right).

This resulted in 18 parameter threshold combinations, which are shown in Table 2. For each combination fibers were reconstructed using the FACT (Fiber Assignment by Continuous Tracking) approach as proposed by Mori et al. [45] (Figure 4). For each ROI the mean fractional anisotropy of all voxels contained in that ROI was calculated. The reconstructed fibers were counted, where a valid reconstructed fiber was defined by a continuous path in the eigenvector field that possesses a seed voxel in a given region-of-interest and conforms to the preset tractography parameter thresholds.

**Table 2:** Tested DTT parameter threshold combinations.

Combination	1	2	3	4	5	6	7	8	9	10	11	12	13	14	15	16	17	18
**FA**	0.1	0.2	0.3	0.1	0.2	0.3	0.1	0.2	0.3	0.1	0.2	0.3	0.1	0.2	0.3	0.1	0.2	0.3
**ANGLE (°)**	20	20	20	30	30	30	45	45	45	20	20	20	30	30	30	45	45	45
**LENGTH (mm)**	25	25	25	25	25	25	25	25	25	50	50	50	50	50	50	50	50	50

Because of the variable ROI sizes for each subject due to the de-normalization process the resulting fiber count was normalized to 1 by dividing the number of reconstructed fibers of a ROI by the voxel count of this ROI. A reconstructed fibers lateralization index was calculated by (FC_naff_-FC_aff_)/(FC_aff_+FC_naff_), where FC is the normalized count of reconstructed fibers, aff corresponds to affected (non-dominant for healthy subjects), naff to non-affected (dominant), respectively. The reconstructed fibers lateralization index has a range of −1 to 1, where index<0 indicates a difference in regards to more reconstructed fibers in the affected/non-dominant hemisphere, index = 0 no difference between hemispheres and index>0 a difference in favor of the unaffected/dominant hemisphere.

### Statistics

Paired t-statistics were conducted for mean FA values of the ROIs of the posterior limb of the internal capsule for affected/non-affected hemispheres in patients and for left/right hemispheres in healthy subjects respectively.

A three-factor repeated measures ANOVA was calculated with the independent variables FA (the three different fractional anisotropy values), ANGLE (the three different angles between two contiguous tracking steps) and LENGTH (minimum reconstructed fiber length 25 mm, 50 mm) and the dependent variable that is the reconstructed fibers lateralization index. If the sphericity assumption was violated in Mauchly's sphericity test, the Huynh-Feldt correction coefficient epsilon was used to establish degrees of freedom. For interactions and main effects post-hoc paired comparisons testing (simple main effects analysis) took place. They were corrected using the Sidak approach.

Additionally, paired t-tests were conducted to determine the parameter threshold combinations that showed the most effective differences between hemispheres. To identify the combination with the largest effect Cohens' d was used to calculate the effect size.
